# Design and Evaluation of Self-Nanoemulsifying Drug Delivery System of Flutamide

**DOI:** 10.4103/0975-1483.76413

**Published:** 2011

**Authors:** Jyothi B Jeevana, K Sreelakshmi

**Affiliations:** *Institute of Pharmaceutical Technology, Sri Padmavathi Mahila Viswavidyalayam (Women’s University), Tirupati, Andhra Pradesh - 517 502, India*

**Keywords:** Dissolution rate, droplet size, self-nanoemulsification, SNEDDS, zeta potential

## Abstract

Flutamide (FLT) is an antiandrogen drug for the treatment of prostate cancer. It has the drawback of poor water solubility and needs enhancement of its dissolution rate in simulated gastric fluids. Hence, it is prepared as self-nanoemulsifying drug delivery systems (SNEDDS) with an aim to enhance its dissolution rate. The objectives of the study are to develop SNEDDS of FLT and to characterize for particle size, self-nanoemulsification, and dissolution enhancement. Solubility of FLT was determined in various oils, surfactants, and cosurfactants. Sesame oil was selected as an oil phase, Tween 20 as surfactant, and PEG400 as cosurfactant due to their higher solubilization effect. Various formulations were prepared by simple mixing followed by vortexing. From studies, the optimized SNEDDS formulation was composed of FLT (8.04% w/w), sesame oil (24.12% w/w), Tween 20 (53.38% w/w), and PEG400 (14.46% w/w). The selected SNEDDS could be self-emulsified without precipitation upon simple mixing. The mean particle size of the SNEDDS was 148.7 nm and percent drug content was 99.66. The dissolution rate of FLT from SNEDDS was faster and higher in three different dissolution media such as 2% sodium lauryl sulfate (97.85%), simulated gastric fluid (0.1 N HCl containing 0.5% Tween 20) (95.71%), and simulated intestinal fluid (pH 6.8 buffer) (96.21%).

## INTRODUCTION

The advent of combinatorial chemistry and high-throughput screening has resulted in the rapid identification of many highly potent new chemical entities. Coincident with the increasing use of these technologies, however, has been a developing trend toward the identification of lead compounds with good therapeutic importance, but fail to elicit their maximum therapeutic effects because of poor aqueous solubility. While these attributes conspire to provide optimized drug-receptor binding characteristics, they also tend to result in poor drug solubility and poor membrane permeability characteristics. As solubility and permeability are considered prerequisites to oral absorption, many of these drugs exhibit poor and variable bioavailability.[1] Such drugs may be recognized by a high-dose-to-solubility ratio, and bioavailability is frequently increased by coadministration of food.[2,3]

The oral delivery of such drugs is frequently associated with implications of low bioavailability, high intra- and intersubject variability, and lack of dose proportionality.[4] To overcome such problems, various formulation strategies are reported in the literature including the use of surfactants, cyclodextrins, solid dispersions, micronization, permeation enhancers, and lipids.[5]

Lipid-based formulations such as self-emulsifying drug delivery systems (SEDDS) have been shown to enhance oral absorption of lipophilic drugs.[6] Although the exact mechanisms responsible for this enhanced absorption are not fully known, it is believed that factors including improved drug solubilization, increased membrane permeability, and lymphatic transport may make significant contributions.[7,8]

Self-nanoemulsifying drug delivery systems are isotropic mixtures of oil, surfactant, cosurfactant, and drug that form fine oil-in-water nanoemulsion when introduced into aqueous phases under conditions of gentle agitation.[9] This property renders SNEDDS a good candidate for oral delivery of hydrophobic drugs with adequate solubility in oils or oil/surfactant blends.[10–12]

Because of self-emulsification in the stomach, the drug is presented as small droplets of oil (<5 μm) leading to improved drug dissolution through providing a large interfacial surface area for partitioning of the drug between the oil and GIT fluid.[13] Other advantages include increased stability of drug molecules, and the possibility of administering the final product as gelatin capsules.[6] For drugs subjected to dissolution rate-limiting absorption, SNEDDS presents a possibility for enhancement in both the rate and extent of drug absorption and the reproducibility of the plasma concentration profile.[14–16]

An example of a commercially available SEDDS is cyclosporine, Neoral^®^, resulted in a twofold increase in its bioavailability in humans compared with other cyclosporine formulations.[17] Selection of a suitable self-emulsifying formulation depends upon the assessment of the solubility of the drug in various components and the droplet size distribution of resultant emulsion following self-emulsification.[10]

Flutamide is a nonsteroidal antiandrogen and chemically it is 2-methyl-*N*-[4-nitro-3 (trifluoromethyl) phenyl] propanamide.[18] In humans, FLT has low oral bioavailability due to poor wettability and low aqueous solubility, poor permeability, and rapid first pass hepatic extraction.[19] Thus, we have proposed to formulate a lipid-based system of FLT to enhance its dissolution rate to achieve optimum oral bioavailability. The main objectives of the study were to develop and evaluate an optimal SNEDDS formulation of FLT.

## MATERIALS AND METHODS

FLT was purchased from M/s Unichem Laboratories Ltd., Raigad, India. Gelatin capsules, No. 0, were kindly supplied by KAPL, Bangalore. All other chemicals, Tween 80 (polyoxyethylene sorbitan monooleate), Tween 20 (polyoxyethylene sorbitan monolaurate), and PEG 400 were obtained from Merck, Mumbai. Span 80 (sorbitan monooleate), span 20 (sorbitan monolaurate), and propylene glycol were obtained from S.D. Fine-chemicals Limited, Mumbai. All the other chemicals were of analytical grade.

### Solubility studies

The solubility of FLT in various oils, surfactants, and cosurfactants was determined as given in Table 1. An excess amount of FLT was added into each vial containing 10 mL of selected vehicle. Then, the mixture was heated at 40 °C in a water bath to facilitate the solubilization. Mixing of the systems was performed using a cyclo mixer (CM 101, Remi, India) for 10 min in order to facilitate proper mixing of drug with the vehicles. Then, the formed suspensions were shaken for 48 h in a mechanical shaker (Remi, India). After reaching equilibrium, the mixtures were centrifuged at 2500g for 20 min to remove undissolved FLT, followed by filtration through a 0.45-μm millipore membrane filter paper. The supernatant was taken and diluted with methanol, and FLT dissolved in various vehicles was quantified by a validated HPLC method (Shimadzu HPLC, class VP series, Japan) with two CC-10 AT VP pumps and class VP software.

**Table 1:** Solubility of flutamide in various vehicles at 25 °C (*n* = 3)

Vehicle	Solubility (mg/mL)	% Solubility (w/v)
Sesame oil	0.912 ± 0.008	91.2
Olive oil	0.623 ± 0.027	62.3
Sunflower oil	0.53 ± 0.031	53.0
Tween 80	0.692 ± 0.018	69.2
Tween 20	0.905 ± 0.035	90.5
Span 20	0.55 ± 0.053	55.0
Span 80	0.438 ± 0.017	43.8
Polyethylene glycol 400	0.747 ± 0.041	74.7
Propylene glycol	0.71 ± 0.05	71.0

### Preparation of SNEDDS formulations

On the basis of the “Solubility studies” section, the oil (sesame oil), surfactant (Tween 20), and cosurfactants (PEG 400) were selected due to their greater solubility enhancement effect on FLT. Various formulations were tried as shown in Table 2. The formulations were prepared by dissolving FLT (8.04% w/w) in the mixture of oil, surfactant, and cosurfactant and were heated at 50 °C in an isothermal water bath. This mixture was mixed well and subjected to vortexing using cyclomixer (Remi, India), until a transparent preparation was obtained. All the mixtures were stored at ambient temperature for further use.

**Table 2:** Composition of self-nanoemulsifying drug delivery systems formulations of flutamide

Ingredients (% w/w)	F1	F2	F3	F4	F5
Flutamide	8.04	8.04	8.04	8.04	8.04
Sesame oil	38.85	36.32	33.59	29.75	24.12
Tween 20	39.83	41.79	44.25	48.76	53.38
PEG 400	13.28	13.85	14.12	13.45	14.46

### Characterization and evaluation of SNEDDS

#### Self-emulsification and precipitation assessment

In brief, various compositions were categorized on the basis of clarity and apparent stability of the resultant emulsion. Visual assessment was performed by dropwise addition of the preconcentrate (SNEDDS) into 250 mL of distilled water taken in a glass beaker at room temperature. The contents were gently stirred either using glass rod or magnetically at ~100 rpm. They were observed immediately after dilution for assessment for self-nanoemulsification efficiency, appearance (transparency), phase separation, and precipitation of drug.

Precipitation was evaluated by visual inspection of the resultant nanoemulsion after 24 h. The formulation were then categorized as clear (transparent or transparent with bluish tinge), nonclear (turbid), stable (no precipitation at the end of 24 h), or unstable (showing precipitation within 24 h).

#### Emulsion droplet size analysis/particle size determination

The selected formulation, F5 that indicated self-emulsification and nonprecipitation was subject to droplet size analysis. The formulation, 100 μL was diluted to 250 mL in a beaker and gently mixed using a glass rod. The resultant emulsion was then subjected to particle size analysis using Malvern zetasizer (Malvern, UK). All studies were repeated for six times, with good agreement being found between measurements.

#### Percent drug content estimation

Flutamide from preweighed SNEDDS was extracted by dissolving in 20 mL methanol. FLT content in the methanolic extract was analyzed using UV-Visible spectrophotometer (Systronics) at 306 nm, against the solvent blank.

#### Zeta potential determination

The zeta potential ζ of the diluted SNEDDS formulations was measured using a Malvern zetasizer (Malvern, UK). The SNEDDS were diluted with a ratio of 1:2500 (v/v) with distilled water and mixed for 1 min using a magnetic stirrer. Zeta potential of each SNEDDS was determined in triplicate.

#### In vitro drug release studies

SNEDDS of FLT was filled in size “0” hard gelatin capsules and used for drug release studies. The *in vitro* drug release studies were also conducted for plain FLT for the comparison of results. The quantitative *in vitro* release test was performed using USP dissolution apparatus II (Elecrtolab, India). The paddles were rotated at 75 rpm. Dissolution studies were run using 900 mL of three different media such as 2% sodium lauryl sulfate, simulated gastric fluid (0.1 N HCl containing 0.5% of Tween 20), and simulated intestinal fluid (pH 6.8 buffer). No enzymes were added to the media. The temperature was set at 37 ± 0.5 °C. During the release studies, a 5-mL sample of medium was taken out and estimated for drug content using UV-Visible spectrophotometer (Systronics) at 306 nm against the solvent blank. The removed volume was replaced each time with 5 mL of fresh medium to maintain sink conditions. The samples were withdrawn at 10, 20, 30, 40, 50, and 60 min. Each study was conducted in triplicate.

## RESULTS AND DISCUSSION

### Solubility studies

Solubility studies were performed to identify suitable oily phase, surfactants, and cosurfactants for the development of SNEDDS of FLT. Because an important consideration when formulating a self-emulsifying formulation is avoiding precipitation of the drug on dilution in the gut lumen *in vivo*.[6] The components used in the system should have high-solubilization capacity for the drug, ensuring the solubilization of the drug in the resultant dispersion.

**Figure 1:**
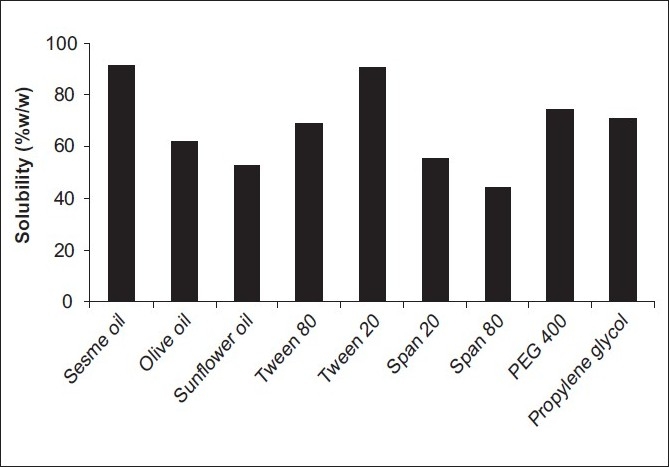
Solubility of FLT in various vehicles at 25 °C (*n* = 3)

The results of solubility studies are reported in Table 1 and Figure 1. It is evident from the results that, among oils, sesame oil (0.912 ± 0.008 mg/mL) exhibited the highest solubilization capacity for the drug FLT, and among surfactants, Tween 20 (0.905 ± 0.035 mg/mL) showed the highest solubility followed by PEG 400 (0.747 ± 0.041 mg/mL) among cosurfactants. Hence, for the preparation of SNEDDS, sesame oil, Tween 20, and PEG 400 were chosen as an oil, surfactant, and cosurfactant.

### Preparation of SNEDDS for FLT

Several SNEDDS systems with the ability to dissolve 50 mg of FLT were prepared and compared. During preliminary study, some SNEDDS were eliminated due to detection of oil droplets on the surface of the diluted SNEDDS, which translates to an incomplete emulsification. SNEDDS that were not able to self-emulsify upon mixing with water under mild-agitation or yielded an unstable emulsions were rejected. A few SNEDDS formulations were eliminated due to the formation of milky emulsions upon dilution. The transparency of the diluted SNEDDS reflects the proximity of the droplet size to that of the nanoemulsion range. Formulations, F1-F5 which were obtained transparent were given in Table 2, and they were subjected to test for self-emulsification and precipitation assessment.

### Self-emulsification and precipitation assessment

Evaluation of self-nanoemulsifying properties of SNEDDS formulations was performed by visual assessment as reported.[20] These studies were carried out on various SNEDDS formulations. During the study, it was found that some formulations, F1 and F2 showed turbidity, precipitation and thus was not stable, due to the relative decrease in surfactant concentration and the presence of PEG 400. Hence, F3, F4, and F5 were prepared with increased concentrations of surfactant. Formulation F5 could be mixed with sesame oil, Tween 20, and PEG 400 and hence was selected as good formulation and subjected to further investigation regarding droplet size, Zeta potential, etc.

### Evaluation of SNEDDS for droplet size analysis, zeta potential, and drug content determination

Droplet size distribution following self-nanoemulsification is a critical factor to evaluate a self-nanoemulsion system. The mean globule size of selected SNEDDS formulation F5, of FLT was 148.7 nm [Table 3] is indicated the ability of the present technology to produce nanoemulsion that offers larger interfacial surface area required for drug absorption.[21,22] An increase in the ratio of the oily phase (sesame oil) resulted in a proportional increase in particle size, because of the simultaneous decrease in the s/cos proportion. Increasing the s/cos (surfactant to cosurfactant) ratio led to decrease in mean droplet size. The optimized SNEDDS, with the highest proportion of surfactant (53.38% w/w Tween 20) at a fixed amount of oil (24.12% w/w), was produced lowest mean particle diameter of 148.7 nm. This could be attributed to an increased surfactant proportion relative to cosurfactant.

**Table 3:** Evaluation parameters of self-nanoemulsifying drug delivery systems formulation of flutamide, F5 (*n* = 3)

Evaluation parameter	Results
Mean droplet size (nm)	148.7 ± 2.325
Mean zeta potential (mv)	-28.7 ± 4.62
% Drug found (mg mL^-1^)	99.66 ± 5.2

The optimized SNEDDS showed high absolute zeta potential value of -28.7 mv. The emulsion stability is directly related to the magnitude of the surface charge.[23–25] Generally, an increase of electrostatic repulsive forces between nanoemulsion droplets prevents the coalescence of droplets. On the contrary, a decrease of electrostatic repulsive forces will cause phase separation. The results of zeta potential and drug content estimation are indicated in Table 3. The percent drug content (99.66 ± 5.2) of SNEDDS of FLT was found satisfactory.

### *In vitro* drug release study

**Figure 2:**
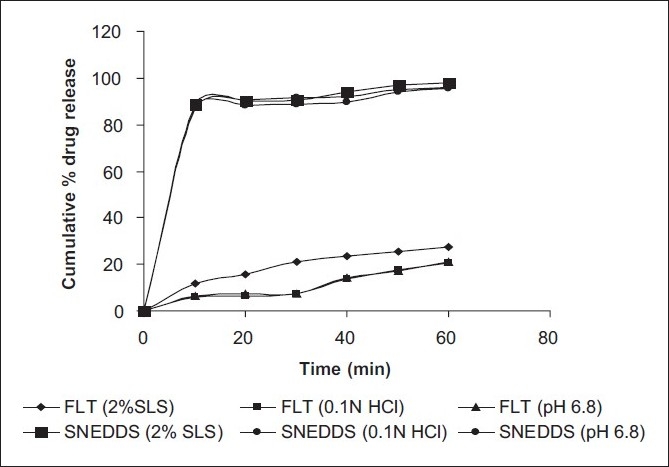
Comparative results of drug release from plain FLT and the SNEDDS formulation in different dissolution media. FLT, Plain flutamide; SNEDDS, self-nanoemulsifying drug delivery system

To understand the characteristics of drug release from SNEDDs, an *in vitro* release study was carried out for pure drug and SNEDDs of FLT in different dissolution media such as 2% sodium lauryl sulfate, simulated gastric fluid (0.1 N HCl containing 0.5% of Tween 20), and simulated intestinal fluid (pH 6.8 buffer). The dissolution profiles of pure drug and SNEDDS formulation are shown in Figure 2. As evident from the drug release profiles, the pure drug evidenced meager solubility of 27.48% in 60 min in 2% SLS, 20.7% in 0.1 N HCl containing 0.5% of Tween 20 and 20.94% in pH 6.8 buffer. The drug release from SNEDDS was markedly high such that 97.85% in 2% sodium lauryl sulfate, 95.71% in simulated gastric fluid (0.1 N HCl containing 0.5% of Tween 20) and 96.21% in simulated intestinal fluid (pH 6.8 buffer). The results indicate instantaneous and remarkably high dissolution of FLT in all three media compared to the pure drug. This higher and faster dissolution rate of FLT from SNEDDS is expected due to the nanoparticle size range of the particles offering higher interfacial area required for dissolution.

## CONCLUSION

An optimized SNEDDS formulation of FLT consisting of FLT (8.04% w/w), sesame oil (24.12% w/w), Tween 20 (53.38% w/w), and PEG 400 (14.46% w/w) was successfully developed with an increased solubility and dissolution rate. The SNEDDS of FLT possessed mean nanoparticle size of 148.7 nm and other ideal characteristics required for enhanced dissolution rate. Thus, our study confirmed that the SNEDDS formulation can be used as a possible alternative to traditional oral formulations of FLT to improve its dissolution rate leading to enhanced bioavailability.
